# The epidemiology of primary headaches in patients with multiple sclerosis

**DOI:** 10.1002/brb3.1830

**Published:** 2020-12-08

**Authors:** Long Wang, Juan Zhang, Zi‐Ru Deng, Mei‐Dan Zu, Yu Wang

**Affiliations:** ^1^ Department of Neurology The First Affiliated Hospital of Anhui Medical University Hefei China

**Keywords:** headache, meta‐analysis, multiple sclerosis, prevalence, systematic review

## Abstract

**Objective:**

Recent studies have shown a pathophysiologic link between headache and multiple sclerosis (MS), but the prevalence of primary headaches among patients with MS differs substantially across studies. This meta‐analysis aimed to comprehensively gather available evidence to estimate the prevalence of primary headaches among patients with MS.

**Method:**

We systematically searched the electronic databases including PubMed, Embase, and Scopus for cohort, case–control, cross‐sectional studies that measured the prevalence of headache among patients with MS. Two reviewers independently screened titles and abstracts to identify the eligible studies and the full texts of the included studies were reviewed. Newcastle‐Ottawa Scale (NOS) was used to assess the risk of bias of the included literatures. We then conducted a meta‐analysis using Stata Software 15.0 to calculate the pooled prevalence of headaches among patients with MS and assess the source of heterogeneity.

**Results:**

We identified 16 eligible studies covering a total of 3,560 patients with MS. The pooled estimated prevalence of primary headaches among patients with MS was 56%. The statistical heterogeneity was moderate with *I* ^2^ of 82.1% (*p* < .001). Both a visual inspection of the funnel plot and Egger’ regression tests revealed no significant publication bias (*p* = .44). The pooled estimated prevalence of migraine (55%) was higher in comparison with that of tension‐type headache (20%). The prevalence of migraine subtype was 16% and 10% for migraine without aura and migraine with aura, respectively. The pooled prevalence of primary headache in case–control group (57%) was approximately in line with the cross‐sectional group (56%).

**Conclusion:**

The overall prevalence of primary headaches among patients with MS was considerably high. Clinical screening of headache among patients with MS will be helpful to formulate an individualized treatment plans and alleviate the physical and mental impact of the disease.

## Graphical abstract

This meta‐analysis aimed to comprehensively gather available evidence to estimate the prevalence of primary headaches among patients with MS. The overall prevalence of primary headaches among patients with MS was considerably high. Clinical screening of headache among patients with MS will be helpful to formulate a individualized treatment plans and alleviate the physical and metal impact of the disease.

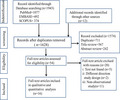

## 1 INTRODUCTION

Multiple sclerosis (MS) is a chronic and progressive demyelinating disease of the central nervous system (CNS), which is characterized by spatially and temporally multiple lesions of unknown etiology (Hauser & Cree, 2020). Epidemiological studies have shown an increasing prevalence of MS in overall in Europe, Latin America, Middle East, and the Mediterranean Basin (Benito‐León & Bermejo‐Pareja, 2010; Cristiano et al., 2016). On the other hand, recent studies have shown that comorbid diseases such as stroke, epilepsy, and headache can adversely affect the quality of life, treatment outcome, and life expectancies in MS patients (Moisset et al., 2013).

Headache is a common neurological comorbidity in MS. The prevalence of primary headaches among patients with MS has been reported to be high, ranging from 35.5% to 70%, with migraine and tension‐type headache being the most frequent types (Busillo et al., 2014; Foley et al., 2013; Moisset et al., 2013). An autopsy of MS patient demonstrated a lymphatic follicular structure proliferation leading to an inflammatory response of the brain meninges (Koshihara et al., 2014). T‐cell and B‐cell activation in meningitis has been presumed to contribute to the high prevalence of headache, especially migraine headache, in patients with MS (Levy, 2009; Moreno et al., 2018). On the other hand, both headache and MS are associated with the functional or structural alterations of brainstem and cortex (Bourgeais‐Rambur, Beynac, & Villanueva, 2019; Tortorella et al., 2006), suggesting a shared anatomical basis for the comorbid relationship between headache and MS.

The prevalence of both migraine and MS is highest in white race, followed by black race, and finally yellow race (Applebee, 2012). However, epidemiologic evidence from current studies showed low consistence in primary headache prevalence among patients with MS. Therefore, we systematically reviewed and meta‐analyzed the available studies to investigate the overall prevalence of primary headaches among patients with MS and provide guidance for screening and diagnosing the primary headaches comorbid with MS in clinical practice.

## 2 METHODS

Our meta‐analysis was conducted on the basis of the Preferred Reporting Items for Systematic Reviews and Meta‐Analyses (PRISMA) guidelines (Moher, Liberati, Tetzlaff, & Altman, 2009).

### 2.1 Search strategy

We conducted a systematic review and meta‐analysis of research articles to assess the prevalence of primary headaches among patients with MS. Two investigators (Long Wang and Juan Zhang) independently searched published articles indexed in PubMed, Embase, and Scopus database from inception to 31 December 2019. Text words and Medical Subject Headings (MESH) terms were used for primary headache and MS “(headache OR migraine OR tension‐type headache OR cluster headache OR trigeminal‐autonomic cephalalgias OR primary headache) AND (multiple sclerosis OR MS OR demyelinating disease).”

### 2.2 Eligibility criteria

We included observational studies which fulfill the following inclusion criteria: (a) study design being cohort, case–control, or cross‐sectional study; (b) diagnosis of primary headaches such as migraine (with or without aura), tension‐type headache, cluster headache being based on the criteria of the International Classification of Headache Disorders (ICHD)‐2 or‐3 (Headache Classification Committee of the International Headache Society, 2004; Olesen, 2018); (c) diagnosis of MS being based on the McDonald or Poser's criteria (Polman et al., 2005; Poser et al., 1983), and patient information obtained through questionnaires or clinical interviews; 4) the prevalence of primary headaches among patients with MS being determined from studies published in the English language. We excluded cases series, letters without original data, duplicate studies, reviews, or studies that did not provide data on the odds or risk of headache in subjects with MS.

### 2.3 Data extraction and quality assessment

One reviewer extracted the data using a standardized data collection form and the information was checked by another reviewer. The following information was extracted from the included studies: surname of the first author, year of publication, country of study, study design, sample size, age of patients, duration of MS, diagnostic criteria, prevalence of headache. The Newcastle‐Ottawa Scale (NOS) was used to assess the quality of the studies (Stang, 2010). We evaluated the following three areas: selection, comparability of studies on the basis of the design, and methodological quality. The maximum score is 9 points. We defined scores of 0–3, 4–6, and 7–9 as low, moderate, and high quality of the included studies, respectively.

### 2.4 Statistical analysis

Data analysis was performed using Stata 15.0 software (StataCorp, College Station, Texas, USA). *I* ^2^ was calculated to quantify the heterogeneity among included studies, A value of *I* ^2^ between 0% and 25% represents absence of heterogeneity; 25% ≤*I* ^2^ < 50%, low heterogeneity; 50% ≤*I* ^2^ < 75%, moderate heterogeneity; *I* ^2^ ≥ 75%, substantial heterogeneity (Higgins, Thompson, Deeks, & Altman, 2003). The random‐effect model for meta‐analysis was used to pool the overall prevalence of primary headaches among patients with MS. Funnel plot and Egger's tests were used to evaluate publication bias. We performed subgroups analysis by headache classifying and study designing to check the robustness of the overall results.

## 3 RESULTS

### 3.1 Characteristics of included studies

**FIGURE 1:**
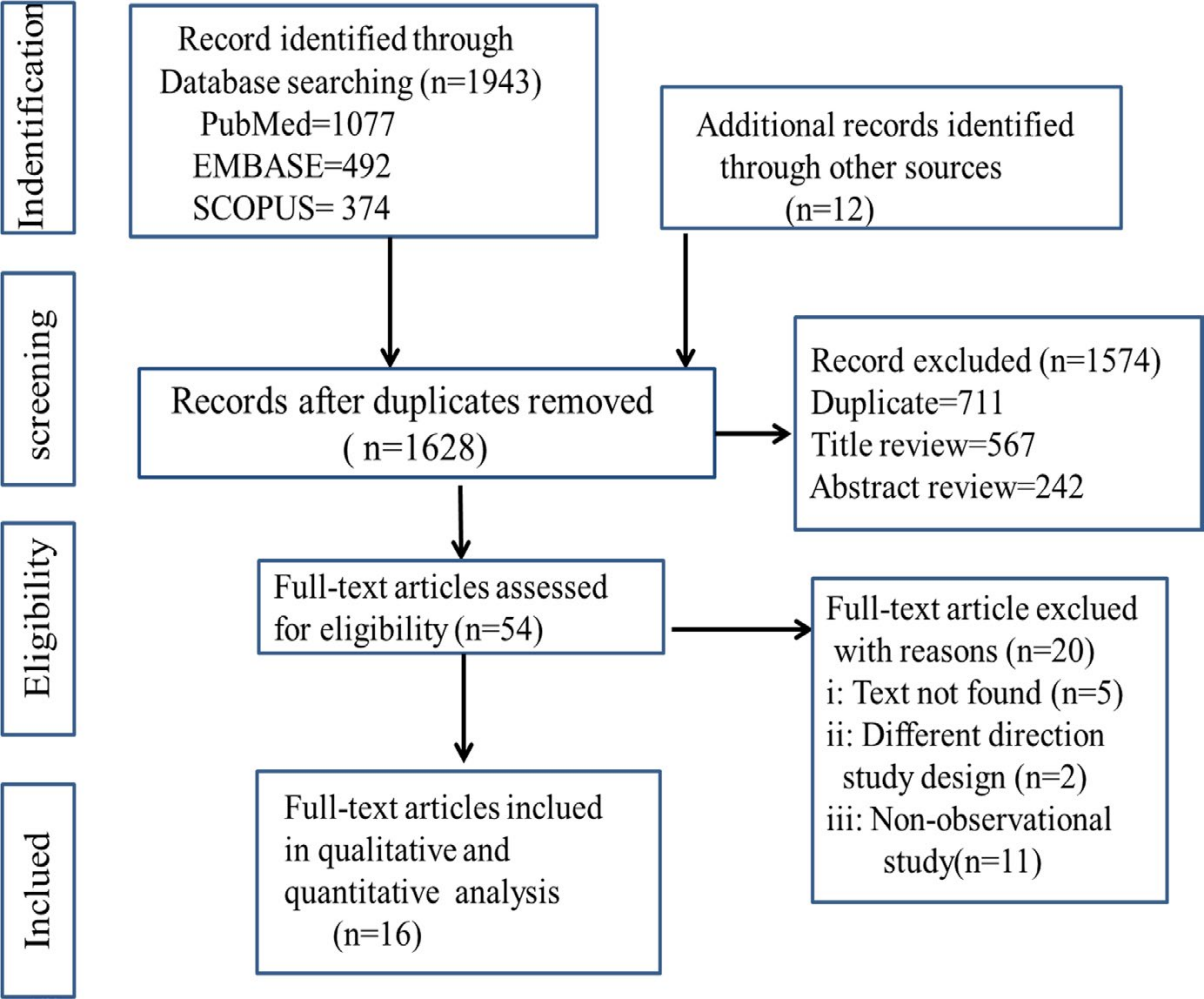
Flowchart of study identification for meta‐analysis

As illustrated in Figure 1, we retrieved a total of articles from our database search. After abstract screening and manual searching of references, we search out 62 articles meeting the criteria for full‐text reviewing. Among the 62 articles, 46 were excluded and 16 research articles were included in the final meta‐analysis (Beckmann & Türe, 2019; Busillo et al., 2014; D'Amico et al., 2004; Doi et al., 2009; Gebhardt, Kropp, Hoffmann, & Zettl, 2018; Gustavsen et al., 2016; Katsiari, Vikelis, Paraskevopoulou, Sfikakis, & Mitsikostas, 2011; Kister et al., 2010; Möhrke, Kropp, & Zettl, 2013; Nicoletti et al., 2008; Özer, Ergün, & İnan, 2018; Putzki et al., 2009; Sorgun, Yucesan, & Yasemin, 2013; Srivastava, Wang, Ugurlu, & Amezcua, 2016; Vacca et al., 2007; Villani et al., 2008).

The characteristics of the included studies are shown in Table 1. Of the 16 articles, reporting a total of seven case–control studies, nine were cross‐sectional studies. Most studies were carried out in Europe and North America. For classification of primary headache among patients with MS, migraine and tension‐type headache were evaluated in all sixteen studies, migraine with or without aura in five studies.

**TABLE 1:** Information about the included studies

First author, year	Country	Study design	Sample size	Mean age of MS (years)	Duration of MS (years)	MS diagnosis criteria	Headache diagnosiscriteria	Prevalence of any primary headaches (%)	Prevalence (%)				NOS score
									MH	MOA	MA	TTH	
Beckmann, 2019	Turkey	Cross‐section	754	36.0 ± NA	NA	McDonald	ICHD‐2	40.5	26.8	18.1	8.7	13.7	8
Özer, 2018	Turkey	Cross‐section	100	33.9 ± 9.0	5.7 ± 4.1	McDonald	ICHD‐2	62.0	46.0			16.0	7
Gebhardt, 2018	Germany	Cross‐section	50	30.0 ± NA	NA	McDonald	MSQ	61.3	47.0			14.3	8
Gustavsen, 2016	Norway	Case‐control	510	50.7 ± 12.5	18.4 ± 11.6	McDonald	ICHD‐2	52.4	18.2			12.7	8
Srivastava, 2016	USA	Cross‐section	233	44.4 ± 12.4	11.9 ± 10.4	McDonald	ICHD‐2	49.0	36.0			5.0	8
Busillo, 2014	Italy	Cross‐section	167	45.0 ± 12.0	12.5 ± 11.0	McDonald	ICHD‐2	56.3	39.5	22.8	16.7	16.8	6
Möhrke, 2013	Germany	Cross‐section	180	43.9 ± 13.1	12.3 ± 8.8	McDonald	ICHD‐2	54.4	16.7	8.9	7.8	12.8	7
Sorgun, 2013	Turkey	Case‐control	139	37.0 ± 10.2	6.7 ± NA	NA	ICHD‐2	66.9	30.9			36.0	7
Katsiari, 2011	Greece	Case‐control	48	37.8 ± 11.9	7.5 ± 4.8	McDonald	ICHD‐2	50.0	22.9			27.1	9
Kister, 2010	USA	Case‐control	204	45.0 ± 12.0	12.5 ± 11.0	McDonald	ICHD‐2	64.2	46.1			18.1	9
Putzki, 2009	Switzerland	Case‐control	491	45.3 ± NA	11.5 ± NA	McDonald	ICHD‐2	56.2	24.6			37.2	9
Doi, 2009	Japan	Cross‐section	127	33.4 + 13.3	11.8 + 9.2	Poser	ICHD‐2	50.4	20.4	16.5	3.9	30.0	6
Nicoletti, 2008	Italy	Case‐control	101	33.6 ± 10.8	10.1 ± 3.0	Poser	ICHD‐2	57.4	19.8			27.7	9
Villani, 2008	Italy	Cross‐section	102	38.7 ± 9.5	8.8 ± 7.6	McDonald	ICHD‐2	53.9	44.1			8.8	6
Vacca, 2007	Italy	Case‐control	238	40.0 ± NA	NA	McDonald	ICHD‐2	51.3	35.7			7.1	7
D'Amico, 2004	Italy	Cross‐section	116	40.6 ± 11.6	13.4 ± 9.4	McDonald	ICHD‐2	57.7	25.0	16.4	8.6	31.9	6

Abbreviations: ICHD‐II, The International Classification of Headache Disorders 2nd edition; MA, Migraine with aura; MH, Migraine Headache, MOA, Migraine without aura; MSQ, Migraine Screening Questionnaire; TTH, Tension‐type headache.

### 3.2 Quality of included studies

NOS was used to assess the quality of the studies. Of 16 included studies, 13 studies are of high quality (NOS score *≥* 7), 3 studies moderate quality (NOS score between 5 and 6), and 0 study low quality (NOS score < 5) (Table. 1).

### 3.3 The prevalence of primary headaches among patients with MS

**FIGURE 2:**
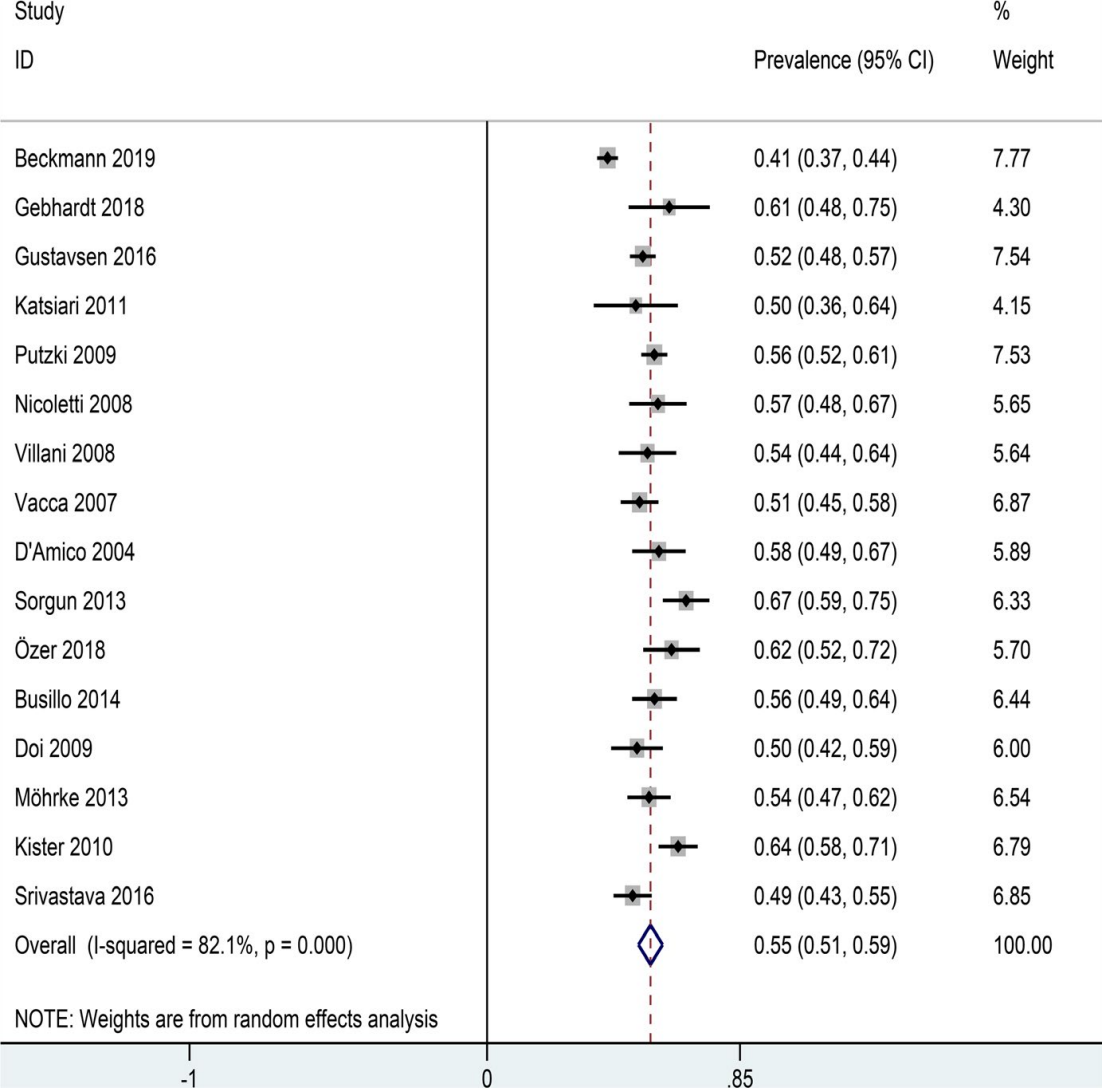
Forest plot for prevalence of primary headaches in patients with MS

The overall prevalence of primary headaches among patients with MS was 55% (95%CI: 0.51–0.59). Nevertheless, obviously heterogeneity among included studies was showed in our meta‐analysis (*I* ^2^ = 82.1%; *p* < .001, Figure 2).

### 3.4 Subgroup analysis

**FIGURE 3:**
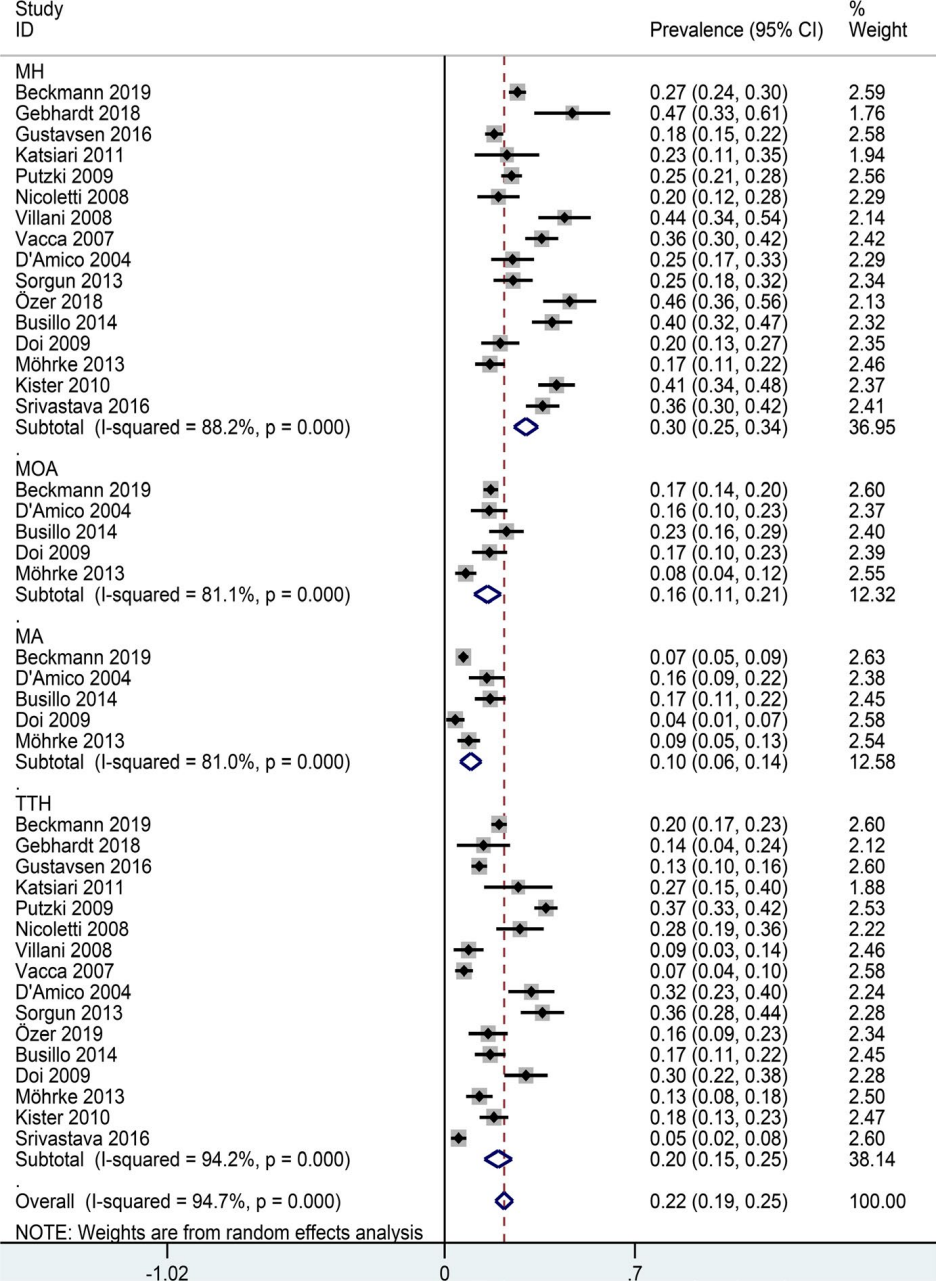
Forest plot for subgroup analysis (types of headache)

We performed a subgroup analysis using of headache types as a moderator. Results showed pooled prevalence of migraine headache (MH) and tension‐type headache (TTH) was 30% and 22%, respectively (Table 2). Five studies reported MA or MOA prevalence in patients with MS and showed prevalence of headaches was 16% and 10%, respectively. (Figure 3).

**TABLE 2:** Subgroup and sensitivity analysis for the prevalence of headaches in patients with MS

Subgroups	Included studies	Prevalence (%)	95% CI	Heterogeneity (*I* ^2^, *Q* and *p*‐value)		
				*I* ^2^ (%)	*Q*‐value	*p*‐value
Types of headache		30				
Migraine	16	30	0.25–0.34	88.2	127.46	0.007
Migraine without aura	5	16	0.11–0.21	81.1	21.21	0.002
Migraine with aura	5	10	0.06–0.14	81.0	21.06	0.002
Tension‐type headache	16	22	0.19–0.25	94.2	259.79	0.010
Study design						
Case–control	7	57	0.53–0.62	68.5	19.04	0.004
Cross‐sectional	9	53	0.47–0.59	81.6	43.51	0.000
Regional distribution						
Europe	12	55	0.53–0.58	31.6	16.08	<0.001
Asia	4	55	0.41–0.69	93.8	48.23	0.019

**FIGURE 4:**
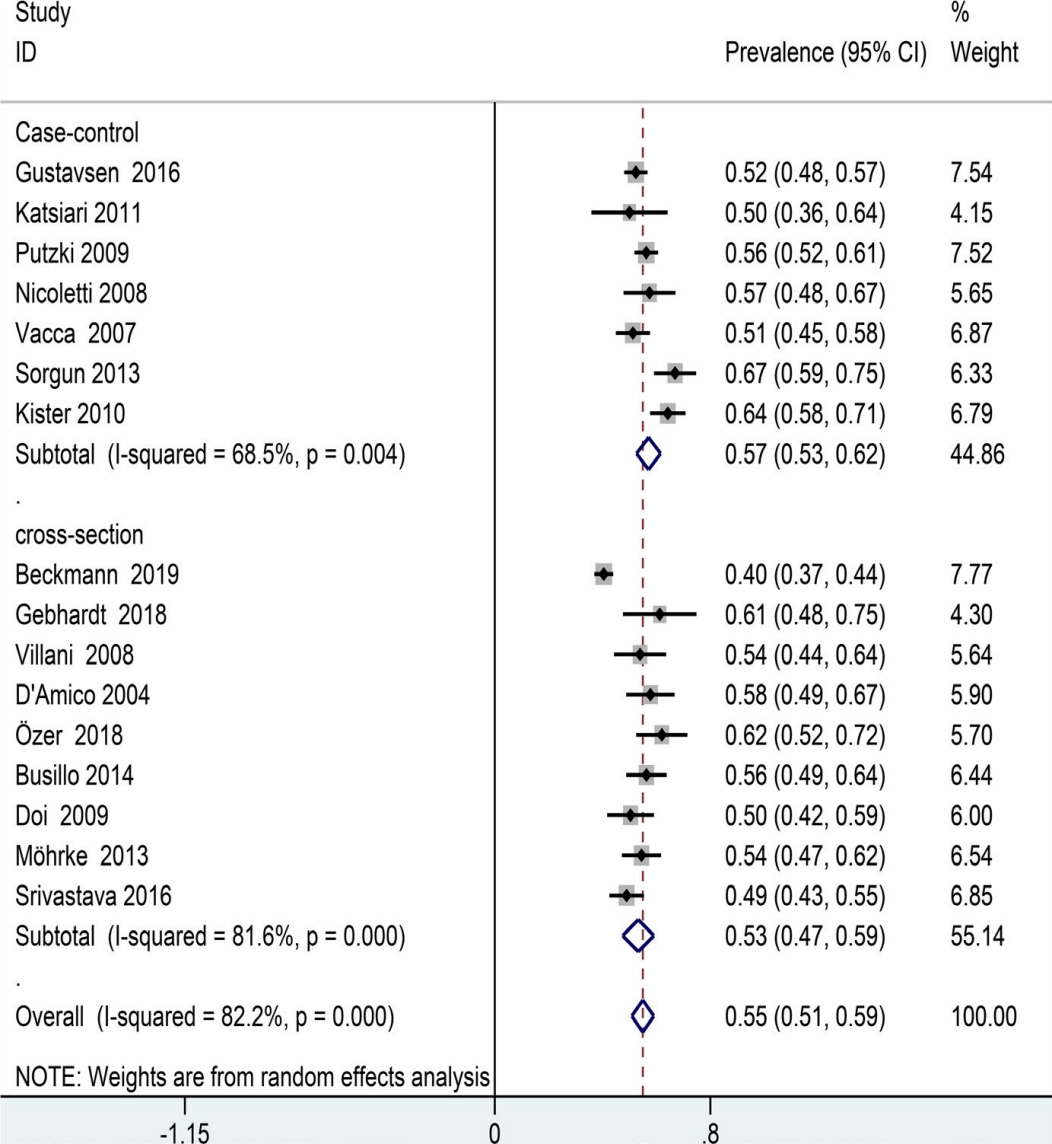
Forest plot for subgroup analysis (study design)

We further conducted a subgroup analysis using the design method as a moderator. The prevalence of primary headaches was slightly higher in the case–control study (57%) than cross‐sectional study (53%) (Figure 4).

**FIGURE 5:**
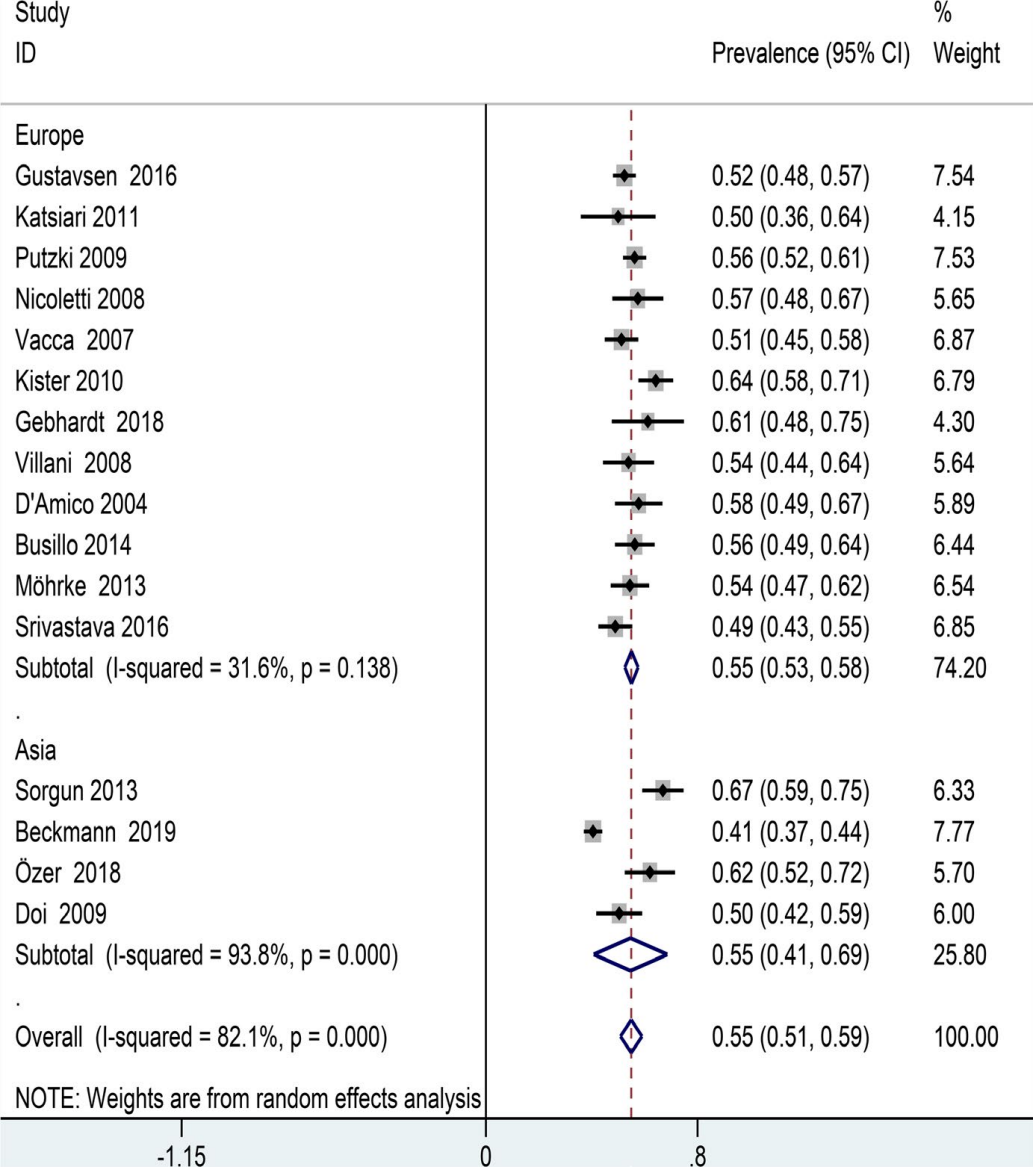
Forest plot for subgroup analysis (regional distribution)

We also conducted a subgroup analysis using the geographical distribution of participants as a moderator. The overall prevalence of primary headaches among patients with MS was 55%, which did not differ from patients in Asian and European countries. The heterogeneity of the included studies was mainly due to the prevalence of primary headaches in Asia (*I* ^2^ = 31.6%; *p* = .019) rather than in Europe (*I* ^2^ = 93.8%; *p* < .001) (Figure 5).

### 3.5 Publication bias

**FIGURE 6:**
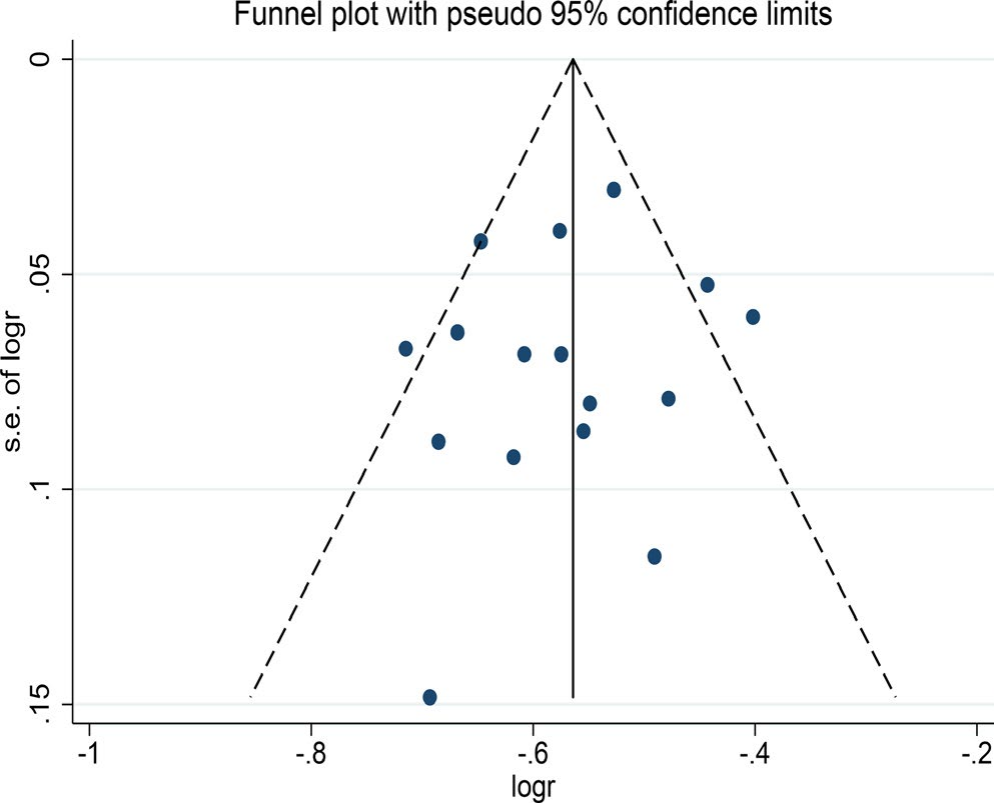
Funnel plot for prevalence of primary headache in patients with MS

A visual inspection of the funnel plot revealed no significant publication bias, and a consistent conclusion was indicated by Egger’ regression tests (*B* = 1.41, *SE* = 1.79, *p* = .44, Figure 6).

## 4 DISCUSSION

In recent years, the comorbid condition of MS has attracted much attention as emerging evidence indicates that comorbidity is related to the delay of diagnosis, quality of life, course of disease, and progression of disability (Fromont et al., 2013; Marrie et al., 2010, 2015). Headache is a common comorbid condition in MS. However, previous studies vary widely in the prevalence of headaches among patients with MS.

We report, for the first time, the prevalence of primary headaches in patients with MS after conducting systematic review and meta‐analysis of globally available studies. Our study showed that the pooled prevalence of primary headaches in patients with MS (57%) was higher than that of WHO report in 2016 in which the overall primary headache prevalence among MS patients was 50% (World Health Organization, 2016). A reasonable explanation for the discrepancy might be due to the differences of study population and designed methods, as MS is 2 to 3 times more common in women than in men and migraine more common in women than in men in the classification of primary headaches (Artero‐Morales, González‐Rodríguez, & Ferrer‐Montiel, 2018; WenJuan, WeiWei, & Xia, 2017). In our included studies, the gender composition of 72% female patients and 28% male patients gives rise to a female/male ratio of 2.6:1. Therefore, this may increase the prevalence of headaches in patients with MS.

Epidemiological evidence suggested that migraine is most closely related to MS, followed by tension‐type headache (La Mantia & Prone, 2015; Moisset et al., 2013). Nevertheless, tension‐type headache was ever reported to be a most common comorbidity in patients with MS, but there was less evidence to support it (Doi et al., 2009). In our review, the overall prevalence of migraine and tension‐type headache among patients with MS was 27% and 10%, respectively. This indicates that it is common that migraine often co‐occurred with MS. Some studies have shown that migraine commonly presented as an initial symptom in patients with MS, indicating that migraine may be a risk factor for MS (Urits et al., 2019). The underlying mechanism is not clear. It seems that the location of plaques among patients with MS was closely associated with an increased occurrence of migraine (Gee, Chang, Dublin, & Vijayan, 2005). Most common localizations of MS lesions are brainstem and cortex associated with migraine pathophysiology. Previous studies demonstrated that 40%–50% MS patients with headache symptom have either cortical or brainstem lesions (Beckmann & Türe, 2019; Mazhari, 2016). MRI study in patients with MS revealed that demyelination within the periaqueductal gray matter (PAG) are several times more likely to have migraine comparing to those with normal PAG (Gee et al., 2005). Further, inflammation‐mediated cortical demyelination can accelerate cortical spreading depression (CSD) which is also the pathological basis of migraine (Husain, Pardo, & Rabadi, 2018; Merkler et al., 2009; Möhrke et al., 2013). These may be suggested that comorbidity of MS and migraine have a common anatomical basis.

Some case descriptions also indicate an association between MS and another common primary headache, cluster headache (Donat, 2012; Mijajlović, Aleksić, & Covičković Šternić, 2014; Pelikan, McCombe, Kotylak, & Becker, 2016). In our included studies, only D'Amico et al. reported that the prevalence of cluster headache among MS is 0.8% (D'Amico et al., 2004). Studies showed that typical symptoms of cluster headache presents while demyelinating lesions are distributed in the entry area of the trigeminal nerve root in the pons (Gentile, Ferrero, Vaula, Rainero, & Pinessi, 2007).

For the subgroup analysis, the pooled prevalence of primary headache in case–control group (57%) was in line with that in cross‐sectional group (56%). This result ruled out bias due to the inclusion of different study design types on the pooled prevalence of headaches among patients with MS. However, the heterogeneity was presented in these subgroups as bias may occur due to the headache data collection through questionnaire or interview in some studies and bias may occur due to the selection of MS patients and controls. Furthermore, the overall prevalence of primary headaches among patients with MS was 55%, which did not differ between patients from Asia and European countries. These may suggest that the risk of primary headaches among patients with MS is similarly common regardless of geography and economic development. In addition, we found high heterogeneity in the prevalence of headaches patients with MS in Asia counties (*I* ^2^ = 93.8) compared to that in European countries (*I* ^2^ = 31.6). Further analysis revealed that few literatures included were from Asia, and three of them were from Turkey. The heterogeneity may be in part due to geographical differences.

The strength of our review and meta‐analysis is the high methodological quality of most of the included literatures. In addition, sensitivity analysis verified the stabilization of our study results. However, some limitations need to be noted. Firstly, majority of the included studies were from European countries, which may decrease the accuracy of estimation. Secondly, we did not investigate the ethnic data due to absence of adequate information. Thirdly, nonstandardized data collection tools were used in some studies and this may affect the pooled prevalence of primary headaches.

## 5 CONCLUSION

We found that the overall prevalence of primary headaches in patients with MS was higher comparing to that in general population. Therefore, we suggest that clinical screening of headache among patients with MS will be helpful to formulate an individualized treatment plan alleviating physical and mental impact of the disease on patients.

## CONFLICT OF INTEREST

The authors report no conflict of interest concerning the materials or methods used in this study or the findings specified in this paper.

## AUTHORS’ CONTRIBUTIONS

LW, ZD, YW: conceptualization. LW and JZ: data curation. LW: formal analysis and funding acquisition. LW, JZ, and MZ: investigation, methodology, resources, and validation. RZ and YW: project administration. LW, ZD, MZ: software. LW, JZ: supervision, writing—review, and editing. LW, JZ, and ZD: writing—original draft.

### Peer Review

The peer review history for this article is available at https://publons.com/publon/10.1002/brb3.1830.

## Data Availability

All data generated or analyzed data in study are included in this article.
